# Surgical Observations; Being a Quarterly Report of Cases in Surgery, Treated in the Middlesex Hospital; in the Cancer Establishment; and in Private Practice. Embracing an Account of the Anatomical and Pathological Researches in the School of Windmill Street

**Published:** 1817-02

**Authors:** 


*Surgical Observations; being a Quarterly Report of Cases in Surgery, treated in the Middlesex Hospital; in the Cancer Establishment; and in Private Practice. Embracing an Account of the Anatomical and Pathological Researches in the School of Windmill Street.* By CHARLES BELL. 8vo. pp. 135, (in future to be pp. 92). London, 1816.

THAT the Medical Officers of Public Hospitals, Fleets, and Armies, possess facilities in the application of remedies, and means of thoroughly investigating the nature of disease, superior to those which the private practitioner can ever hope to enjoy, is an evident truth; and this superiority is still more evident in surgery than in physic. The private practitioner may persuade his patient to swallow this or that remedy; but an operation, or other painful remedial measure is seldom submitted to, till the last extremity; and then, perhaps, under a different surgeon, so that the chain of connection between cause and effect; between origin, progress, and termination of morbid structure or function is broken, and all useful deductions lost. This is not the case in the Establishment above alluded to, or in the service of his Majesty by sea or land. Here submission on the part of the patient to even the most arbitrary measures of the medical superintendant is a *sine qua non*; and consequently, the phænomena of disease and the effects of remedies lie open to observation, undisguised by the delusive representations and imaginary feelings of morbid sensibility, effeminacy, hypochondriacism, luxurious refinements, and other aberrations from Nature, which characterise civilized society. The officers of public medical establishments, however, have almost always been averse to giving publicity to their experience in those excellent theatres for observation, whereby the medical community has suffered much. The confinement of this kind of practical knowledge to the immediate agents or spectators has probably been comparatively disadvantageous to the parties themselves. The more extended and varied trials which points of practice, emanating from such sources, must undergo, in consequence of their diffusion throughout the medical world at large, would very generally either confirm, improve, or entirely overthrow them; but this salutary reaction of the public voice is not called forth, except in an indirect and partial manner, when the results of hospital practice come garbled before the public from the pens of ci-devant students, who too often endeavour to construct a temporary monument of their fame from the *disjecta membra* of their quondam masters.

We hail therefore, with pleasure, the prospect which Mr. Bell offers in these Quarterly Reports. A man, in every respect so eminently qualified to act, observe, and record, may and must confer a lasting obligation on the profession by a conscientious and diligent discharge of the duty he has here imposed on himself.

That a host of jealous, envious, and malignant *defamers* will lie in wait to traduce the character, misconstrue the motives, and depreciate the merits of Mr. Bell, for thus giving publicity to his practice in the hospital to which he has been lately appointed, he cannot but be aware. Happy are we that he has spirit and good sense to brave the arrows of calumny which impotent rivalry will now level at him from every quarter. It behoves him, however, where the Argus eyes of criticism are eager for a flaw, to be extremely circumspect in his conduct, and especially to give his adversaries no cause for charging him with mercenary motives in the publication under review. We would hope and believe, that upon this occasion,

Tanto major FAMÆ sitis est quam pecuniæ.

And therefore we would humbly recommend, that neither by *platter nor price,* our author prevent the middling and inferior classes of the profession from sharing in the fruits of this experience, or give cause to the higher ranks of stigmatising the work as a *job* founded solely on lucrative and interested motives.

Before entering on our analytical labour, we would just observe, that the results of hospital practice are seldom applicable in their full extent to the exigencies or routine of the private physician or surgeon. The student goes hot into the country from the great theatres of anatomy and surgery in the metropolis, fraught with powerful remedies, sharp instruments, and firm resolutions to eradicate and subdue every ill that flesh is heir to; nor is he long there, before he meets with *cases* that are exactly adapted to his purpose. The *first* active dose, however, that he administers, will generally be the *last* with that patient, and he will be peremptorily told to send no more of that *stuff* which is fitter for a horse than a Christian! This rebuff will terribly disconcert our young Esculapius; for how shall he now give a fair trial to that invaluable formula of his master Dr. So and So, at the hospital in London? He next meets with a scirrhous tumour, or a scrofulous enlargement of the knee-joint, which has just arrived at that stage of progress wherein excision or amputation may yet give the patient a fair chance of recovery. He candidly states, that any other means than the operation would only be trifling with the life of the patient, and that he cannot conscientiously prescribe medicines where he is convinced they can be of no avail.

"Then, Sir, (says the patient or friend) you may save yourself the trouble of calling any more here, since the *knife* is your only remedy."

Two or three months afterwards, he is called upon by a professional friend, to assist him in performing the same operation, for the proposal of which he himself was previously dismissed, and lost the confidence of the family! These are some of the checks which the hospital student is destined to meet in the first days of his journey through life!* By degrees, they break off the fine edge of candour and honour with which his feelings are pointed, and he finds himself obliged to fight the world with its own unworthy weapons, or suffer himself to pine in indigence and neglect, while ignorance and impudence snatch the bread from his mouth, and vilify his character in private.

---

* These are not ideal representations, or confined to the outset of practice; an instance, of which the above is a literal and faithful transcript, happened to one of the Editors of this Journal within these six months.

These hints are thrown out as a salutary caution to Mr. Bell's pupils not to be too precipitate in the application of their master's precepts, till time shall stamp their own character with the seal of public confidence and personal responsibility.

FIRST REPORT.

It will be in the memory of our readers, that about a twelvemonth ago, Mr. Young's book on cancer announced a new and successful mode of curing that *opprobrium medicorum;* and that trials were to be made of its efficacy in the cancer-ward of the Middlesex Hospital. We ourselves gave it a trial immediately after the publication appeared, but unsuccessfully; we saw two other trials made with the same result; and we had long set it down in our own minds as a bubble that would soon burst and be no more remembered.

The prognostication is now verified by the report of the medical committee of the aforesaid establishment, in which it is stated that eight cases of open cancer, and eight of scirrhus have been submitted to the treatment by compression; some for several months, and others for a shorter period.

"That, in some of the cases of open cancer, combined with considerable œdema, the pressure was useful in lessening the volume of the tumour; but that it had not, even in a single instance, any salutary influence upon the specific nature of the disease. It frequently gave so much pain, that the patients could not, after repeated trials, endure it under any modification whatever, and often it appeared to hasten the fatal event.

"That in the scirrhous tumours, the disease advanced, rendering extirpation necessary in two instances; in six others, the disease passed into ulceration, assuming the usual malignant appearances, and terminating in death. Your committee, therefore, have to lament that compression cannot be regarded as a remedy for cancer."

In a succeeding paragraph, the committee state,

"That they have, in many cases, succeeded in obtaining great alleviation of suffering—such alleviation as might, perhaps, induce more speculative minds, less disciplined by experience, to conclude, that they had at length succeeded in discovering a cure for cancer."

In a long chain of "additional observations" on the inefficacy of compression, by Mr. Bell himself, he nowhere lets fall the slightest notice respecting the means of alleviating—*almost curing* that dreadful and painful disease. Although it is Mr. Bell's intention to treat fully of this complaint in the progress of the work, yet, without anticipating his plan, some hint might here have been dropped, that possibly would pour the balm of mitigation into the breast of many a suffering female.

### Second Report.

### *Diseases and Wounds of the Larynx, &c.*

Mr. Bell first refers to the preparations in his collection, which he liberally opens to the members of the profession. In this collection he points out four examples of laryngeal disease, shewing the nature of the croupy membrane, which is one of coagulable lymph, thrown out by inflammation, and added to the mucous secretion. In fifteen examinations of these parts after death, it has not appeared to Mr. Bell, that the patient was destroyed by the violence of the inflammation, nor the irritation *directly* from the inflamed membrane; but from the presence of this secreted membrane acting as a foreign body, spasming the glottis, obstructing the passage, and confining the mucus. Death is ultimately the consequence of effusion in the lungs, occasioned by the struggle and difficulty. Mr. Bell thinks that Mr. Chevalier's operation* for the relief of a case of this kind, authorises a repetition of the experiment. The surgeon, however, should take care that the operation is performed before effusion has taken place.

*Ulcer within the sacculus laryngis.* This woman had been ill three months with what she terms "asthma and hoarseness;" for which she has had her throat blistered and pustuled by an ointment. At present, short cough; spits half a pint daily; constant dyspnœa, with occasional paroxysms of greater violence than usual; speaks in a whisper, with husky, reedy, feeble sound; pain in the throat and thyroid cartilage, increased on pressure; dysphagia. She died without any struggle.

*Sectio Cadaveris.* Larynx round the chordæ vocales ulcerated, with strings of coagulable lymph crossing the part; a bony portion of the thyroid cartilage necrosed, and hanging loose in the remains of the sacculus laryngis; the passage of the larynx nearly closed by accreted coagulable lymph; marks of chronic inflammation upon the inside of the trachea; effusion in the lungs. We this morning visited a patient who appears to labour under a

---

* *Vide* No. 8 of this Journal.

similar disease, complicated with stricture in the œsophagus. Mr. Bell does not here state the appearance of that tube.

Mr. B. infers, that ulceration of the glottis is a frequent complaint, and fatal, if not remedied. He thinks these ulcerations may be divided into slow chronic, or scrofulous; and venereal. The disease deserves the name of *Phthisis Laryngea,* for it has all the consequences of true phthisis. The glandular structure of the larynx and trachea becomes enlarged; ulcerates, and throws out purulent secretion, destroying the patient more or less rapidly according as the larynx or only the trachea is affected. The attack is gradual at the beginning: there is wheezing; troublesome cough; purulent, sometimes bloody sputum, with painful spasm in the throat; anhelation, and loss of voice. Ultimately, pain in the chest; laborious respiration; effusion in the lungs; death! This we have, in several instances, witnessed; and lately, in an exquisitely marked case.

CASE. *August* 3d, 1815.

Our author was called up in the night to a patient of Dr. Latham's, who was suffocating. She was sitting up in bed, breathing with difficulty, and great noise. Could speak only in whisper; pointed to the throat and pit of the stomach, as the seats of pain. Her breath came whistling as through a reed. The blood was still flowing from the bites of leeches. No history could be obtained. She was exhausted and pale, with suffering, anxiety, and watching; the respiratory organs in such violent operation, that effusion into the lungs must soon take place, if not already. An incision, two inches in length, on the fore-part of the throat; the blood fell upon the breast, leaving the wound clear; the bleeding somewhat relieved her; dissected deep on the fore-part of the trachea, much blood flowiag.

"The sterno-hyoidei and thyroidei muscles were powerfully in motion during each inspiration; and they raised themselves into the appearance of two round columns, and by their starting forwards, gave an unusual depth to the trachea. Every touch of the knife on the front of the trachea brought a flow of blood; and I saw that it would be most inconvenient, if not dangerous, to open the trachea." 24.

Mr. Bell enlarged the wound a very little upwards; laid down the isthmus of the thyroid gland, and exposed the space between the cricoid and thyroid cartilages, through which a sharp bistoury was pushed, when the air in expiration came forcibly and audibly out, spurting the secretion from the hole. The slit was enlarged upwards and downwards, without cutting the cartilages. The slit was then kept open by a spatula, when immediately the harsh sawing sound of the air in the contracted glottis ceased, and the air played easily, with a siffling sound, through the wound. The woman now fell fast asleep, from the previous exhaustion. After some time, Mr. Bell introduced a tube; but the breathing was instantly stopt: a struggle commenced, and the tube was thrown out like a cork from a bottle. As this would not do, the bend or bite of a doubled catheter wire was introduced, and kept the wound sufficiently extended for the purpose of respiration. The poor woman lived seven weeks, breathing through the artificial opening. In the last night of her miserable existence she was seized with great difficulty of breathing, ran frantic about the ward, fell down upon the floor, tore the instrument from her throat, and was suffocated.

*Post Mortem.* A loose spongy granulation sprang from the sacculus laryngis of the left side, and lay completely closing up the chink of the glottis. Beneath this there was a deep ulcer; and within the thyroid cartilage were several ulcers, communicating with which there were abscesses on both lateral faces of the thyroid cartilage. The lungs were free from disease.

This is a very interesting case, and does great credit to Mr. Bell, as it unfolds to us a glimpse of that professional ardour, perhaps enthusiasm, without which few men can ever attain a lasting reputation in medicine or surgery.

### Case 2d. *Ulcer of the Glottis.*

Mary Mellon was admitted into the hospital on the 12th of March, 1815, and died in half an hour afterwards, without a struggle. The day before this, she was walking in the streets at her usual pace. Laryngotomy was performed, and the air rushed out with great force; but life could not be restored. The epiglottis was destroyed by ulceration, and there was a deep foul ulcer within the false glottis. Her friends informed, that fifteen months previously she had sore throat like quinsy, which was not treated as venereal. Before this she could sing, but never afterwards: she had hecking cough and much expectoration.

CASE 3d. *Disease of the Larynx.*

Fanny Murray, ætat. 25, had symptoms similar to M. Mellon's. Mr. B. put his finger into the glottis, and felt it rough. Entered the hospital April 18; had been ill since Christmas. At present her whispers are scarcely audible; she is in imminent danger of suffocation; has not been able to lie down these three nights; expectorates mucus and pus; breathing has a harsh sawing sound.—*Evening.* She was in a most critical state. Mr. Bell made a small pad of lint, attached it to the ring of a catheter wire, and bent the wire so as to pass over the root of the tongue and epiglottis. Then having dipped the lint in a solution of lunar caustic (20 grains to the half ounce) with the fingers of the left hand, he pressed down the tongue, stretching the fore-finger of the same hand over the epiglottis; then directing the wire along this finger, he removed the point of the finger from the glottis, and introduced the pad of lint into the opening, and pressed it with his finger. On withdrawing the lint, instead of coughing, she began to speak more audibly than usual, and experienced neither cough nor spasm from this rough operation. The application was repeated four times, and her breathing was sensibly relieved when he left her.—19th. Touched it again; she is better.—20th. Better; had the feet and legs immersed in the nitro-muriatic bath twice a day.—22d. Has had the acid bath several times, up to near the loins.—26th. Again applied the solution very strong; she is much better.—29th. Is remarkably better; her gums sore.—5th May, Dr. Scott visited her; never saw the mouth so sore from the nitro-muriatic bath.—30th. She is almost well, and some weeks afterwards left the hospital. p. 38.

Mr. Bell next relates some particulars of a case when ulcer in the glottis was imitated in hysteria. He found a young woman lying in a state of torpor; her mouth open, and breathing with a croupy sound, apparently in the act of suffocation. But when he observed that she lay reclined on her back, with the countenance of a natural colour, and the lips vermillioned, without any catch or struggle in the muscles of the chest, he saw that it was hysteria; and next morning she was well. We are at this moment attending a much more remarkable case of this kind, which will probably be related in the Journal of the ensuing month.

*Laryngotomy.*—The necessity for this operation is not very rare; but the proper time for its performance is too often lost, by the apprehension of its formidable nature. It requires, Mr. B. conceives, but a small scalpel to divide the membranous space betwixt the cricoid and thyroid cartilages. This done, the handle is to be substituted for the point of the knife; and turning it round, so as to open the slit, the patient immediately breathes free.

"There is here nothing to alarm the most timid operator. No great turgid veins are opened; he is above the thyroid gland, and above the anastomosing branch of the thyroid arteries. The part is strongly marked by the prominence of the thyroid cartilage above, and the ring of the crycoid cartilage below." 46.

Mr. B. prefers this interspace between the two cartilages to all others. If the cricoid cartilage be cut through, a branch of the thyroid artery is in the way; perhaps the superior lobe of the thyroid gland. A very little lower, and we touch the isthmus of that gland, which will bleed like an artery. If we cut on the fore-part of the trachea, below the gland, we are confined in a narrow space; the trachea is deep; the thyroid veins turgid; the hæmorrhage embarrasses the operator, and if it flows into the trachea, may suffocate the patient. To cutting out a piece of the cartilage Mr. B. objects, because it commits more injury than appears necessary; makes an orifice which requires a process of granulation to fill up; and gives facility for granulations to project into the larynx afterwards and obstruct the canal. 48.—Laryngotomy, however, offers but a temporary relief. If the original obstruction (whether it be aneurism of the carotid, disease of the thyroid gland, a foreign body lodged in the pharynx or larynx, or any other cause) continue, the patient will sink, from the morbid influence being propagated to the lungs, and disturbing their office.

### *Case of Scirrhous Contraction of the Pharynx.*

May 3d, 1816, Jane Nichols has been in the hospital about three weeks ago, and is now returned. She had then dysphagia with pain; a hard tumour could be felt projecting from the larynx into the bag of the pharynx. Leeches, blisters, emetic, course of Plummer's pill; considerable amendment was the result. She can give no history of her complaint. At present, dysphagia; sense of something rising from the throat towards the ear, from pressure of the diseased part against the Eustachian tube.

"When I put my finger into her throat, I can distinctly feel the epiglottis; and pushing the finger beyond this, I can distinguish the left side of the false glottis to be irregular and tuberculated; but I think not ulcerated. Still further back I can feel a tumour projecting into the pharynx, having its root on the back part of the layrnx."

A tube was attempted to be passed into the œsophagus to convey the food; but it produced too much irritation. Six leeches to the neck; the sides of the throat to be blistered alternately; to smoke strammonium; the bowels to be kept open with Plummer's pill.

May 10th. She is worse in every respect. With the fore-finger of the left hand, Mr. B. presses down the tongue, and draws it forward; with the fore-finger of the right hand, sugar and calomel are rubbed on the rough diseased surface; from this she feels relief, and always solicits a repetition of the operation; but the benefit appears to be little more than the lessening of morbid irritation.

29th of May. The calomel has made her mouth sore, without any other amendment—omitted. A small caustic issue instituted near each upper horn of the thyroid cartilage. For some time after this she got worse, and was once near suffocation; but on the *23d of June*, " she is much better in all respects, and has undoubtedly been greatly benefitted by the issues." These promising appearances, alas, were only fallacious gleams. The disease made progress towards the glottis, though the passage to the stomach was still kept permeable; and after great suffering, she expired on the *18th of July*.

*Sectio Cadaveris.* The seat of the disease was in the membrane of the pharynx, from whence it had spread to the œsophagus on one side and larynx on the other; firm, white, scirrhous tumours studded, and nearly closed, the beginning of the œsophagus and the pharynx. On looking through the larynx from below, two white tumours were seen projecting from the sides of the tube, nearly closing the avenue to the lungs.

*Substance of Mr. Bell's Remarks on the foregoing Cases.*—The whole alimentary canal is subject to diseases of the above description. The scirrho-contracted rectum is an example. The scirrhous thickening of the pharynx or œsophagus may be much relieved by the bougie and frequent application of leeches to the side of the throat. The caustic issues in the same place, and the bougie will effect a cure where there is a mere thickening by common inflammation or scrofulous action; but where carcinomatous disposition exists, it is evident, that all relief must be transitory. The caustic, though ultimately advantageous, sometimes increases the spasm at first; in the same manner as when it is applied to urethral stricture.

We shall pass over Mr. Bell's description of a preternatural bag formed by the membrane of the pharynx, as not containing matter of much practical importance.

*Stricture of the Œsophagus cured by Caustic.*—W. H. ætat 40, tall, thin, and apparently healthy, was suddenly seized in May last with dysphagia; next day he experienced the same difficulty, which continues with little variation to the present time. He is now thin and weak from absolute want; he does not suffer from sickness, nor has he any retchings so common in similar cases; no sense of suffocation, distention of stomach, or borborygmus. Slime and mucus collect in his throat, and are discharged with such morsels as he cannot swallow: liquids only can be swallowed. Any thing solid, like a crust of bread, works its way up again. On the introduction of a bougie, an obstruction was discovered behind the cricoid; he is easier since. Next day the dysphagia has returned. Three or four applications of caustic so much relieved him, though the bougie was still obstructed, that he left the hospital.

Mr. Goolden, of Maidenhead, next communicates to our author the case of a woman, *Drusilla Champ,* who laboured under stricture of the œsophagus during twenty-four years, and died of it at last. The stricture was situated at the lower part of the pharynx opposite to the cricoid cartilage, and would only admit a horse-hair or bristle to pass.

The next case is apparently one of stricture, but very different in reality. John Terry, ætat. 70, was admitted into the hospital unable to swallow either solids or liquids, and pointed to the cricoid cartilage as the seat of obstruction. He had uneasiness about the stomach, and suffered much from hunger. Tongue foul; bowels constipated; slept well. Has been only two months ill. The dysphagia came on suddenly; and once or twice suddenly disappeared. An œsophagus bougie was passed into the stomach without meeting any obstruction. He died.

*Sectio Cadaveris.* The œsophagus was præternaturally enlarged and distended where it enters the posterior mediastinum. On opening it, a large irregular tumour presented, soft, spongy, and pendulous from the inner coats of the tube. The preparation is engraved, and forms the third plate.

*Mr. B's Remarks.* The feelings and expressions of the patients in these cases are often deceptive. Inflammation will produce stricture; stricture will produce pain; pain inflammation, and inflammation increase of stricture: hence the disease gets worse and worse, until the canal is almost totally closed. The derangement is in the inner membrane of the tube, and is situated immediately behind the cricoïd cartilage. It is not so much formed by a membranous partition, as by a general and somewhat irregular puckering of the whole membrane of the œsophagus. In the very worst stricture possible, it does not necessarily follow, that ulceration shall take place. The proper remedy is the bougie. Here, as in some cases of urethral stricture, the attempt to dilate will sometimes excite spasm and obstruction, rendering the application of caustic necessary. The bougie should be introduced with a gentle hand; it requires dexterity, but not force. Mr. B. thinks, that common inflammatory sore throat sometimes lays the foundation for stricture in the œsophagus; inflammations of the tube from violence will have this effect. Medical men are apt to apprehend the worst upon all occasions; and thus often to mistake symptomatic derangement for formidable organic disease. Many muscles are concerned in the act of swallowing which are occasionally employed in other functions, or belong to other combinations; hence, they may become deranged individually from their relation to remote parts. The pharynx and œsophagus frequently suffer more from disordered action, not inaptly termed nervous, than from organic injury; generally from disorder of the stomach and bowels. Spasmodic dysphagia, for the most part, takes place at the termination of the lower constrictor pharyngis, or at the termination of the tube in the stomach. Two diseased actions may be mistaken for stricture or scirrhous ulcer of the passage; viz. spasms of the muscular coat, and paralysis; they equally prevent the passage of the morsel. These are symptomatic and transitory; their cause is gastric, hepatic, or uterine. Our author thinks, that when authors speak of spasm continuing for days or during months, they surely mistake paralysis for spasm of the tube. Obstruction from a permanent cause is always attended with more or less of spasm. The remedies in spasmodic œsophageal stricture are such as tend to correct the visceral disorder, and give activity to the gastric and hepatic organs; or such as are more directly antispasmodic. 1. Mercurial friction on the neck. 2. Mercurial pill to salivation. 3. Valerian in draughts; and antispasmodic liniments. 4. Stimulating vapours, as of assafœtida inhaled. 5. Laudanum taken in small quantities, so as to rest in the œsophagus: anodyne glysters, &c. p. 86.

The fourth and last Report in this publication is on *Fistula in Perineo*, illustrated by a dozen or more cases. They more than prove the alarming nature of the sudden bursting of the urethra, and extravasation of urine into the cellular membrane—the necessity of giving the urine immediate issue—of preventing the recurrence of the evil—of soothing the local irritation, and of supporting the system against the influence of the extensive mortification which ensues from the infiltration of the urine. We shall select the second case, as one of peculiar interest, and affording a good specimen of the manner and matter of the work under review.

"*Bursting of the Urethra, Sloughing of the Scrotum, extensiv Sinuses around the Belly.*

"*Tuesday.* A professional gentleman called upon me, and expressed considerable uneasiness on account of an œdematous swelling of the fore-skin and scrotum, which he had observed in one of his patients. On our way, I inquired into the circumstances, and learned that the patient was fifty-five years old, and had spent thirty years in India. On returning to London, he had put himself under a surgeon of eminence, for the cure of strictures in the urethra, and afterwards submitted himself to this gentleman's care, who was at this time in course of dilating the strictures by the use of the bougie. On Sunday he had used a middle-sized bougie, and had passed it without violence into the bladder. No blood followed this introduction of the bougie, but during the night there occurred a very considerable hæmorrhage from the urethra. Next morning a swelling appeared, which was supposed to be extravasated blood, and the following day I was requested to attend. I found the patient lying on his sofa in a state of fever and tremor. He said he had shiverings in the night, and the fever now upon him had succeeded. I observed that the urine came away with difficulty, and required him to strain a great deal. It now only came in drops, although before the attack the stream had been free. The swelling, which had occasioned apprehension, was, indeed, very like œdema; but on the right side and upper part of the scrotum, there was a tumour which pitted, and evidently contained fluid, I had, therefore, no doubt that the appearances were owing to extravasated urine. A moderate-sized gum catheter was passed into the bladder. The parts were fomented, and after the bowels were opened he had an opiate. I requested to be sent for on the slightest change taking place.

"*Wednesday.* I find a very serious change to have taken place. Having a sudden call to make urine, in his agitation, and in drawing out the plug of the catheter, he withdrew the instrument itself, and made water from the urethra. He expressed himself pleased with the large stream, but soon after he felt the swelling of the scrotum materially increased. The scrotum has become generally and greatly distended. I immediately opened the scrotum with the abscess lancet very freely; there came full eight ounces of blood, and watery fluid drained away, so as to occasion a very great reduction of the swelling even while I remained with him. A catheter was introduced, and particular injunctions given. Dr. Babington being expected, I did not prescribe for him at that time.

"*Thursday.* He is better: he has less heat, and the scrotum is diminished. A black spot is on the front of the scrotum. He has a draught with five drops of laudanum every two hours.

"*Friday.* The scrotum is of a dark red colour; a blush of erysipelas extends to the bottom of the belly; the black spot is not larger, but a slough will take place there. The cellular membrane within the incision is white and dead; I have broken down the cellular membrane to give free passage to the fluids. The pulse is calmer; there is not the same degree of tremor, nor is the tongue so dry. He is ordered a draught of decoction of bark, with a few drops of laudanum and diluted sulphuric acid. The fomentation to be extended to the belly.

"*Saturday.* No moisture on the skin; tongue dry; pulse 90; more taciturn. Dr. Babington has approved of more support. A pint of Port wine to be taken, in small quantities, in the course of the afternoon, with soup and jellies.

"*Sunday.* The slough is very extensive, and the testicles will be laid quite bare. I have dissected away a great quantity of the ragged cellular membrane with the forceps and scissars. The wound is dressed with pledgits dipt in camphorated liniment, and the carrot poultice covering the scrotum. Fomentations are continued to the belly. His bowels are moved every morning by clysters.

"*January 3d, Wednesday.* I have now no fear for my patient's life. Pulse 80; skin more moist; he has taken more nourishment and the sloughs begin to separate from the edge of the suppurating skin. I fear the urethra may be included in the slough, and then the case will be lamentable.

"5th. *Friday.* The putrid mass is very large. I dissected off a large portion to-day. The redness on the belly is gone; but a hardness and caking of the skin above the pubis and groin remains. A milky fluid exudes from the integuments; we have urged him to live better.

"8th. *Monday.* The slough is separated, and the testicles entirely uncovered. Already granulations show themselves. The catheter has been withdrawn, and larger ones substituted. This has been an operation of some difficulty, from the length and fullness of the fore-skin. But now that the slough can no longer confine the urine, the catheter is withdrawn, and he makes water through the urethra without moistening the dressings below; and the case being much simplified, I have taken my leave.

"12th. *Friday.* The surgeon in attendance has again requested my opinion; a swelling has taken place round the lower part of the belly. Above each groin there is an abscess with surrounding hardness. As it is thought possible that the urine has again found its way into the cellular membrane, the catheter is introduced; the integuments of the penis are very much distended; the glans penis cannot be felt through them, and it is consequently difficult to introduce the catheter. Pulse 115; skin dry; urine high coloured; no appetite.

" 19th. *Friday.* Since my last note a considerable change has taken place. The inflammation extends around the lower part of the belly, forming a band two hands' breadth in diameter. It has been kept low by cold applications, but a band of hardened integuments incircles the belly, passing from the pubis round both groins and over the alæ iliorum.

" From this date to the 29th there is no note. The abscesses above the groin became soft and ulcerated, while the integuments around were caked and hard. The fluid discharged sunk through all the bed-clothes and mattress. I enlarged the opening, took away some sloughy cellular membrane, and gave vent to eight ounces of pus, thin and without smell. This I did several times, for as the cellular substance was washed down to the opening by the flow of matter, it choked the passage and confined the matter.

" After the sloughs were discharged, the sores were dressed simply, and the sinuses had compresses laid along them, and supported by a flannel roller. The whole surfaces affected were fomented morning and evening, and every attention paid to support the patient's strength.

" *February* 1. Another depôt of matter has formed on the right side. The quantity of thin inodorous matter now flowing from under the integuments of the abdomen, is very great. He stood to-day while I examined him, and the matter poured in streams from ulcers. I have great fears for his life. His pulse is quick; his hands dry; he has a great expectoration, and is very much reduced in strength. What is favourable is, that he has a resolute mind to obey his physician, and is not too much alarmed at parting with life; and that he takes his wine and bark, submits to have the lower bowels emptied by enema, and takes light nourishment.

" 10th. Called to-day, and finding the sinuses sluggish, advised an injection of sulphat of zinc. The experiment to be made cautiously on one of the sinuses.

" *March 28th.* One of the openings still discharges, and I find him still confined. The remaining integuments have drawn themselves about the testicle, so as greatly to conceal the ravages which have taken place. He must be sent out of town to regain force of constitution for the filling up of the sinuses: they have become habitual.

" *April 20th.* Being again called here, I find the sinuses still open, and running all round the belly to the loins. The matter has dropt down upon the scrotum on the right side, where there is red ness and tumour ; I have passed a seton from the opening on the left side of the belly to that above the left groin. I have opened the abscess on the right side of the scrotum, and have given a depending orifice to all the sinuses and ulcers round the right haunch. The sinuses were injected with a solution of zinc, and a more perfect apparatus of compression used. He takes the islandic moss, and milk diet.

" 23d. All the sinuses of the right side amended. The seton on the left side has caused some inflammation, and a purulent discharge with fœtor. I have withdrawn it, and bound down compresses on the tract of the sinus.

" From this time the patient made rapid amendment. The sinuses closed, and he regained his wonted health." P. 96.

The remaining cases we must pass over, referring to the work itself; of which we have given a very full analysis. Although the price appears great for the type and plates [four in number]; yet, when we consider the importance of the matter—the authenticity of the facts—the fidelity of the narratives, and the talents of the writer, we cannot but conscientiously and strenuously recommend the work to the perusal of every practical surgeon, and to the libraries of all classes in the profession.

